# Supplementary material for: Proteogenomic Analysis of Burkholderia Species Strains 25 and 46 Isolated from Uraniferous Soils Reveals Multiple Mechanisms to Cope with Uranium Stress
Source: Cells. 2018 Dec 12;7(12):269. doi: 10.3390/cells7120269 (PMC6315756; doi:10.3390/cells7120269)
Supplement: Supplementary file 1 [file cells-07-00269-s001.pdf]

Supplemental Materials:

Proteogenomic Analysis of *Burkholderia* Species Strains 25 and 46 Isolated from Uraniferous Soils Reveals Multiple Mechanisms to Cope with Uranium Stress

Meenakshi Agarwal [1,†], Ashish Pathak [1,†], Rajesh Singh Rathore [1], Om Prakash [2], Rakesh Singh [3], Rajneesh Jaswal [1], John Seaman [4] and Ashvini Chauhan [1,*]

1. Environmental Biotechnology Laboratory, School of the Environment, 1515 S. Martin Luther King Jr. Blvd., Suite 305B, FSH Science Research Center, Florida A&M University, Tallahassee, FL 32307, USA; meenakshiagarwal.iitb@gmail.com (M.A.); ashishpathak72@gmail.com (A.P.); rajeshrathore854@gmail.com (R.S.R.); jaswal.rajneesh@gmail.com (R.J.)
2. National Centre for Microbial Resource, National Centre for Cell Science, Pune 411007, India; prakas1974@gmail.com
3. Translational Science Lab, College of Medicine, Florida State University, Tallahassee, FL 32304, USA; rakesh.singh@med.fsu.edu
4. Savannah River Ecology Laboratory, University of Georgia, Aiken, SC 29802, USA; seaman@srel.uga.edu

* Correspondence: ashvini.chauhan@famu.edu; Tel.: +850-412-5119; Fax: 850-561-2248

† These authors contributed equally to this work

Received: 5 November 2018; Accepted: 10 December 2018; Published: 12 December 2018

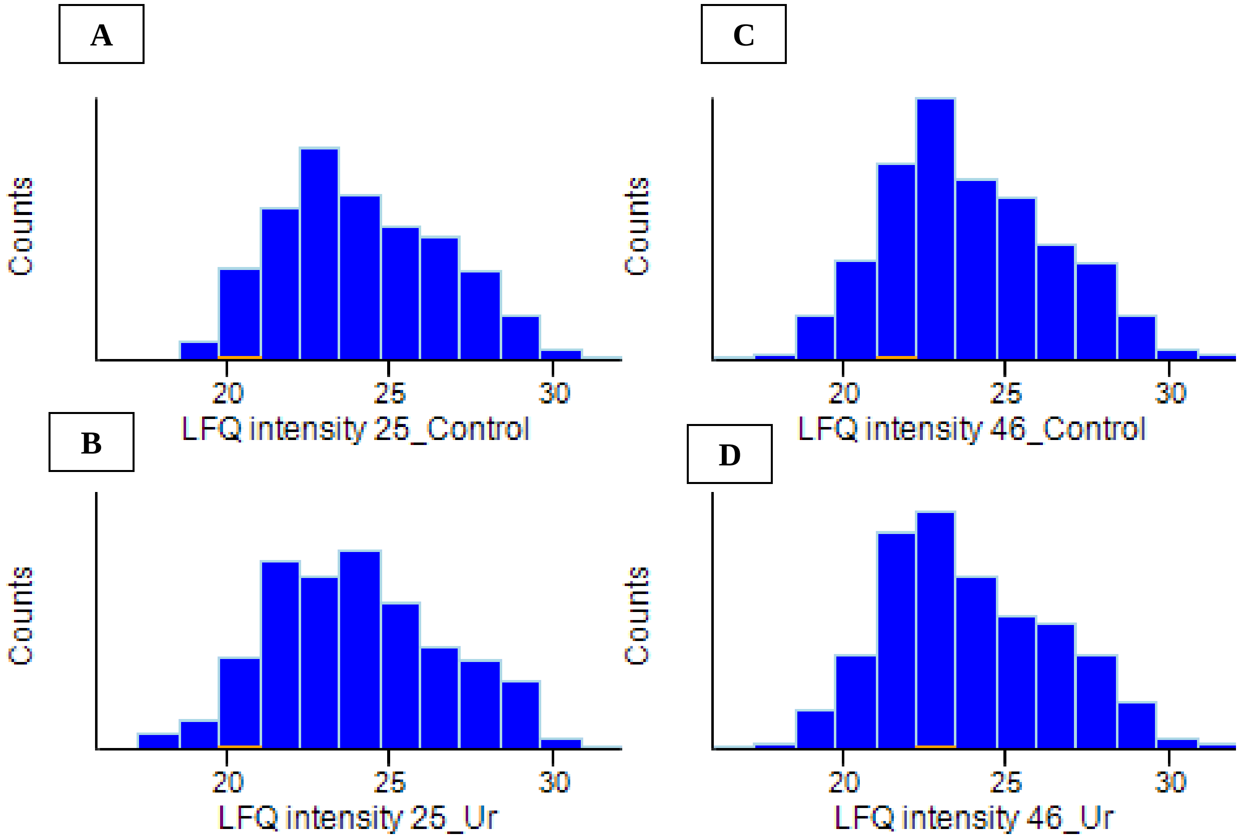

Figure S1. Shown are the label free quantification (LFQ) proteomics data analysed using Perseus software for A and B, strain 25 in the presence and absence of uranium and; C, D, strain 46 in the presence and absence of uranium, respectively. Data were log2(x) transformed and histograms were plotted.

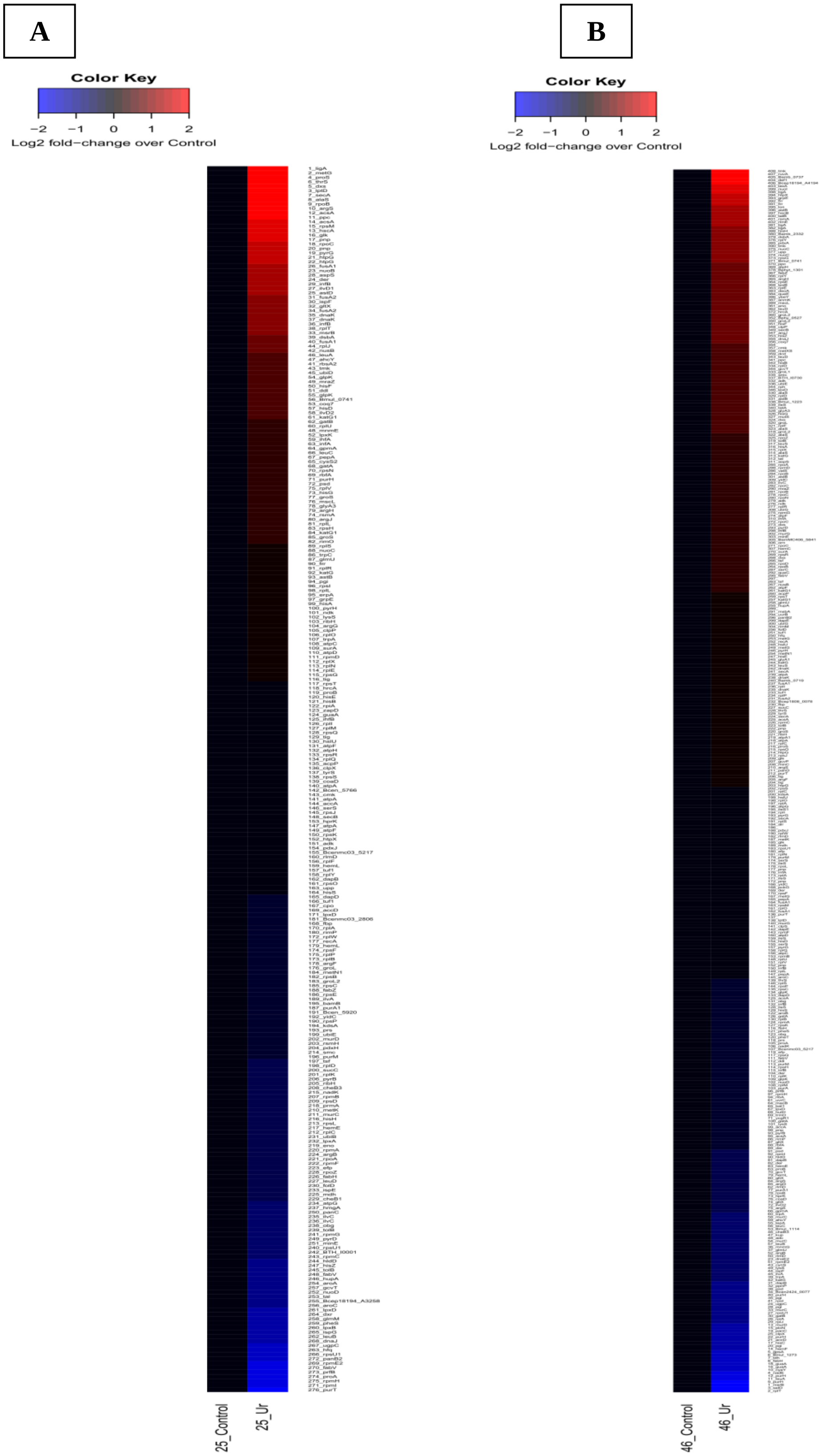
A
Color Key
−2 −1 0 1 2
Log2 fold−change over Control
25_Control
25_Ur
B
Color Key
−2 −1 0 1 2
Log2 fold−change over Control
46_Control
46_Ur

Figure S2. Heat maps from SRS-25 (left) and SRS-46 (right) show the of relative abundance of all the identified proteins between control and uranium amended microcosms. The proteins were sorted on the basis of their fold change value as compared to their control. Gradient of fold change is shown by color code where blue represents the lower most fold change value and red represents the highest fold change value.

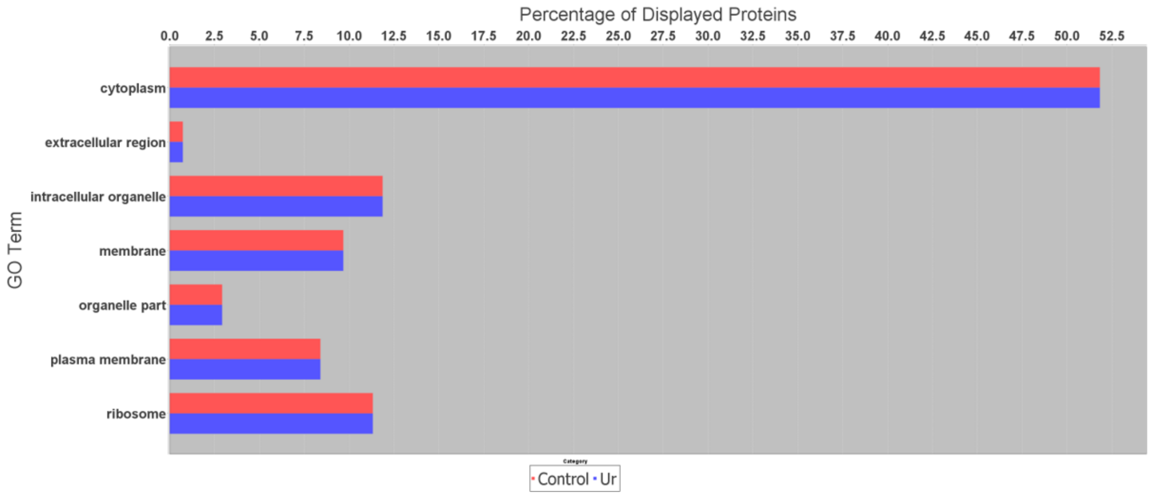

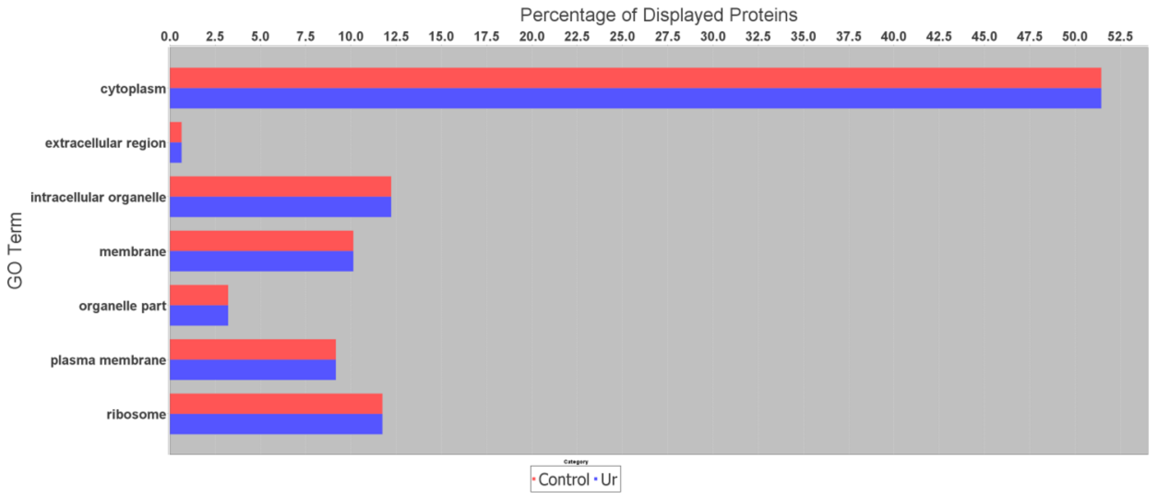

Figure S3. Plotted are proteins obtained from strains SRS-25 (top) and SRS-46 (bottom) with or without uranium exposure, analyzed by Scaffold software using gene ontology (GO) analysis tool.
